# Impaired Fluid Intake, but Not Sodium Appetite, in Aged Rats Is Mediated by the Cyclooxygenase-Prostaglandin E_2_ Pathway

**DOI:** 10.3389/fnagi.2020.00019

**Published:** 2020-02-28

**Authors:** Denovan P. Begg, Andrew J. Sinclair, Richard S. Weisinger

**Affiliations:** ^1^School of Psychology, UNSW Sydney, Sydney, NSW, Australia; ^2^Faculty of Health, Deakin University, Burwood, VIC, Australia; ^3^School of Psychology, La Trobe University, Bundoora, VIC, Australia

**Keywords:** thirst and drinking, water intake, aging, salt appetite, sodium

## Abstract

Aging results in decreased fluid intake following dehydration and other dipsogenic stimuli; similar reductions in sodium intake have also been observed with aging. Given that cyclooxygenase (COX)-derived prostanoids are elevated in aged rats in the midbrain and proinflammatory prostanoids are known to decrease fluid intake in dehydrated rats, the aim of this study was to determine if the reductions of fluid intake and sodium intake in aging are mediated by proinflammatory eicosanoid signaling. Therefore, we examined the effect of acute COX inhibition in adult (4 months-old) and aged (30 months-old) rats prior to ingestive behavior challenges. COX inhibition, using acetylsalicylic acid (ASA), increased fluid intake in aged, but not adult, rats in response to 24-h dehydration. ASA had no effect on salt intake following sodium depletion and ASA did not change basal fluid or sodium consumption in either age group. Hypothalamic COX-1 and -2, prostaglandin E synthase (PGES) and inducible nitric oxide synthase (iNOS) mRNA expression were all elevated in aged animals, leading to elevated PGE_2_ levels. COX expression in the hypothalamus was reduced by ASA treatment in rats of both ages resulting in reduced PGE_2_ levels in aged ASA treated animals. These data indicate that the reduced fluid intake that occurs in aging is due to increased COX-PGE_2_-mediated inflammation. However, the reduced sodium intake in these animals appears to occur *via* an alternate mechanism.

## Introduction

Aging significantly impairs fluid and sodium balance in mammals (Phillips et al., 1984; Thunhorst and Johnson, 2003); these impairments result from alterations to both behaviorally-mediated intake (Thunhorst et al., 2009) and reduced renal function and renin-angiotensin system (RAS) activity (Ledingham et al., 1987; Antunes-Rodrigues et al., 2004). Aged animals have reduced fluid intake following dehydration (Thunhorst and Johnson, 2003; McKinley et al., 2006) and also have reduced sodium intake following peripheral angiotensin converting-enzyme (ACE) inhibition (Rowland et al., 1996; Thunhorst and Johnson, 2003) and sodium depletion (Begg et al., 2012a,b).

Prostaglandins (PG) are produced when cyclooxygenase (COX) enzymes metabolize polyunsaturated long-chain fatty acids to prostaglandin (PG) H; PG synthases then metabolize PGH into active PGs. Suppression of fluid intake in response to dehydration occurs following administration of E series prostaglandins (Goldstein et al., 1979; Pérez Guaita and Chiaraviglio, 1980). Aged rats have increased PGE synthase (Tang and Vanhoutte, 2008) and PGE_2_ in the midbrain (Meydani et al., 1985); these effects are suppressed and drinking behavior restored when animals are fed omega-3 fatty acids (Begg et al., 2012a,b). There are additional changes related to inflammatory signals with aging including an up-regulation of inducible (i) nitric oxide synthase (NOS; Wu et al., 1997; Adachi et al., 2010), a COX activator (Salvemini et al., 1993). Together, these observations may indicate involvement of prostanoids in the reduced fluid intake that occurs with aging.

Non-steroidal anti-inflammatory drugs are commonly used to reduce inflammation caused by PGE_2_ through inhibition of COX enzymes. Therefore, the aim of the current study was to determine whether the reduced fluid and sodium intakes that occur in aging are the result of increased eicosanoid levels, and whether inhibition of COX restores this deficit. Furthermore, given that there are established interactions between inducible nitric oxide synthase (iNOS) and the COX/PGE pathway, mRNA expression of these genes in hypothalamic tissue levels were examined.

## Materials and Methods

### Animals, Drugs and Measurements

**Figure 1:**
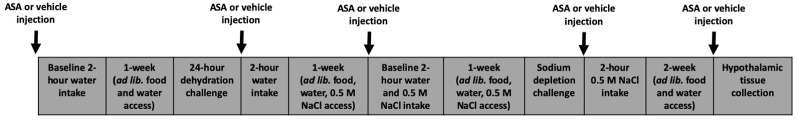
A timeline of the experimental procedures.

Forty-six male Brown Norway rats were bred in the central animal house, La Trobe University. Animals were tested at either 4 months-of-age (adult animals; *n* = 22) or 30 months-of-age (aged animals; *n* = 24). Standard laboratory rat chow and water were available *ad libitum* except during challenges, as noted. Animals were single housed for the duration of the experiment and were maintained on a 12-h light/dark cycle. Acetylsalicylic acid (ASA; Sigma-Aldrich, Saint Louis, MO, USA) was dissolved in 20% dimethyl sulfoxide (DMSO) and water. Injections of ASA were given IP at 50 mg/kg, vehicle injections were 20% DMSO and 80% water. Water and sodium intakes were measured using an automated drinking system as previously described (Begg and Weisinger, 2008). There were 2 weeks between the drinking challenges described and a further 2 weeks prior to animal sacrifice. For experimental timeline, please refer to Figure 1. La Trobe University Animal Ethics Committee approved all animal procedures.

### 24-Hour Dehydration

Baseline 2-h water intakes were established 1 week before testing. Baseline measurement was performed 1 h after ASA or vehicle injection. For dehydration measurements, rats had water removed the morning prior to testing. Twenty-three hour later, animals were injected with ASA or vehicle. Then, 1 h later, drinking water was returned. Cumulative water intake was measured for 2 h, and then food was returned.

### Sodium Depletion

As previously described (Weisinger et al., 2010) rats were provided access to 0.5 M NaCl solution, water and food for 2 weeks prior to sodium depletion. One week prior to sodium depletion, baseline 2-h sodium intakes were measured 1 h after ASA or vehicle injection. On the day of sodium depletion, 0.5 M NaCl solution and food were removed, while access to water remained and animals were administered furosemide (20 mg/kg I.P.; Apex Laboratories, Australia). Twenty-three hour after furosemide administration animals were injected with ASA or vehicle, 1 h later, 0.5 M NaCl solution was returned and intake was measured for a 2-h period. After 2-h, food was also returned.

### Hypothalamic mRNA Expression

Animals were injected with ASA or vehicle and 1 h later injected with pentobarbital sodium (325 mg/kg). Brains were removed, the hypothalamic block dissected and stored at −80°C. RNA was extracted from ~50 mg of hypothalamic tissue (*n* = 7/group) using a standard Tri-reagent technique (Trizol; Ambion, TX, USA), RNA quality was determined using a Nanodrop 2000 spectrophotomer (Nanodrop, DE) and converted to cDNA (SuperArray, MD, USA). Real-Time quantitative PCR was performed as previously described (Begg et al., 2012a,b) using primers for cPLA_2_, COX-1, COX-2, PGES, EP_1_, EP_2_, EP_3_, EP_4_, neuronal (n) NOS, endothelial (e) NOS and iNOS (SuperArray, MD, USA). Relative expression ratios were normalized to β-actin and are expressed relative to adult animals using the 2-ΔΔC_t_ method.

### Hypothalamic PGE_2_ Concentration

Hypothalamic concentrations of PGE_2_ were determined from the hypothalamic block. The supernatant of homogenized tissue was filtered through C18 columns based on previous descriptions and analyzed using ELISA (*n* = 4–5/group; Assay Designs).

### Statistical Analysis

Three-way (age × drug × time) ANOVAs were used to compare the effects of age and COX inhibition on behavioral measures. Two-way (age × drug) ANOVAs were used to analyze gene expression and PGE_2_, *post hoc* comparisons were performed using Fisher’s protected Least Significant Difference tests, significance was accepted at *p* < 0.05, data are reported as mean ± SEM.

## Results

### 24-Hour Dehydration

**Figure 2:**
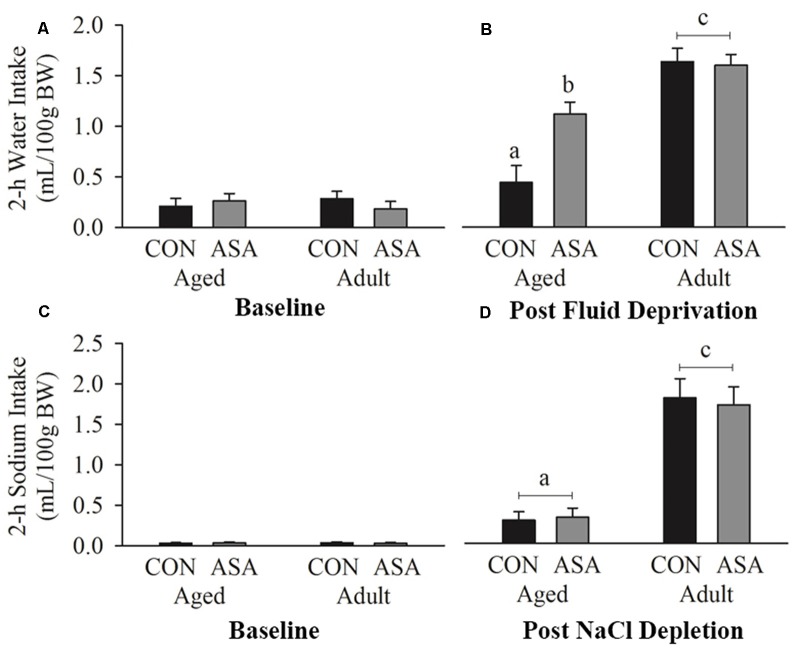
Water intake **(A)** did not differ between groups at baseline, however **(B)** following 24-h fluid deprivation, aged rats (*n* = 24) drank significantly less water relative to young adult rats (*n* = 22). Rats treated with acetylsalicylic acid (ASA) consumed significantly more water than vehicle treated (CON) rats in the aged but not the young adult population. **(C)** 0.5 M NaCl intake did not differ between groups at baseline, however **(D)** following sodium depletion aged rats (*n* = 24) consumed significantly less NaCl than young adult rats (*n* = 22). There was no effect of ASA treatment within the groups. Differences between groups are denoted by different superscript letters.

There were no differences in baseline 2-h water intakes between the groups (Figure 2A). Following 24-h dehydration all groups had increased 2-h drinking responses (*p* < 0.05; Figure 2B). Thirty-months-old rats drank less fluid in the 2 h following fluid return compared with young adult animals (*p* < 0.05). Pre-treatment with ASA significantly increased water intake in 30-months-old but not young adult rats (*p* < 0.05; Figure 2B).

### Sodium Depletion

Baseline intakes of sodium were not different between the groups (Figure 2C). All groups had greater sodium intake following depletion than at baseline (*p* < 0.05), however, 2-h 0.5 M NaCl solution intake was lower in aged rats than in adult rats (*p* < 0.05; see Figure 2D). ASA administration did not alter sodium intake in either young adult or aged rats (*p* < 0.05).

### Hypothalamic mRNA Expression

**Figure 3:**
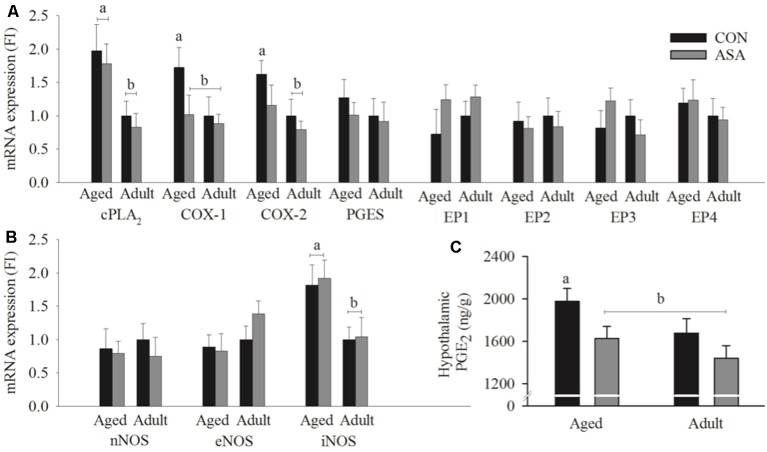
Hypothalamic mRNA expression of **(A)** cPLA2, COX-1 and COX-2 was significantly increased in hypothalamic tissue of aged control rats relative to young adult rats. COX-1 expression was significantly reduced in the ASA treatment group of aged animals. No significant differences were seen between groups in the expression of PGES and EP1–4 (*n* = 7/group). **(B)** There were no significant differences between groups in the hypothalamic expression of nNOS and eNOS, however inducible nitric oxide synthesis (iNOS) mRNA expression in hypothalamic tissue was significantly increased in aged rats relative to young adult rats (*n* = 7/group). **(C)** Hypothalamic PGE2 concentrations in the hypothalamic tissue of aged control rats was significantly increased relative to young adult rats. PGE2 was significantly reduced in the ASA treated group of aged animals. Young adult and aged control rats; *n* = 4–5/group; black. ASA treated rats; *n* = 5/group; gray. Differences between groups are denoted by different superscript letters, the absence of a letter indicates no significant differences.

There was significant up-regulation of mRNA expression of cPLA_2_ (*p* < 0.05), COX-1 (*p* < 0.05), and COX-2 (*p* < 0.05) in aged control animals relative to young adult animals. COX-1 (*p* < 0.05), but not COX-2 or cPLA_2_, was significantly lower by ASA administration in aged animals. There were no differences in EP receptor or PGES mRNA expression related to age or ASA administration (see Figure 3A). mRNA expression of iNOS was significantly increased in aged animals (*p* < 0.05), this was unaffected by ASA treatment. nNOS and eNOS mRNA expression were not affected by age or ASA (see Figure 3B).

### Hypothalamic PGE_2_ Concentration

There was significant increase of hypothalamic PGE_2_ in aged control animals relative to young adult animals (*p* < 0.05). Hypothalamic PGE_2_ was significantly lowered by ASA administration in aged animals (*p* < 0.05; see Figure 3C).

## Discussion

Aging leads to a significant decline in fluid intake in response to dipsogenic stimuli (Silver et al., 1991; Whyte et al., 2004; McKinley et al., 2006) and sodium intake in response sodium depletion and ACE inhibition or sodium depletion (Rowland et al., 1996; Thunhorst and Johnson, 2003). The current study assessed whether the mechanism of this effect was mediated *via* COX-PGE_2_ induced inflammation in the hypothalamus. Following 24-h fluid deprivation ASA treatment increased water intake in aged animals; the effect was not attributable to a generalized effect on water intake, as ASA did not increase drinking in hydrated aged rats. These data are consistent with previous work demonstrating no changes in drinking following COX inhibition in adult rats (Foca et al., 1985).

This study also aimed to establish if the increased prostanoid levels observed in aging also caused the reduced intake of sodium, following sodium depletion. Unlike the observations of water intake, ASA did not increase sodium intake following furosemide injection in aged or young adult rats. These data indicate that the reduced intakes of water following dehydration, and sodium, following depletion, are most likely controlled by separate mechanisms. This study found higher levels of cPLA_2_ in aged animals cPLA_2_ indicating an increased in release of arachidonic acid from cell membrane phospholipids in the hypothalamus which allows for increased metabolism to series 2 prostanoids (Touqui et al., 1986).

Aging resulted in increased mRNA levels of both COX-1 and COX-2 in the hypothalamus. Conversion of arachidonic acid to PGH_2_ occurs *via* the COX-1 or COX-2 enzymes and it has previously been demonstrated that COX-1 mRNA expression is elevated in aging (Aïd and Bosetti, 2007). ASA significantly suppressed mRNA expression of COX in aged rats; similar observations have previously been reported with ASA treatment in models of high COX expression (Trujillo-Murillo et al., 2008). mRNA expression of genes downstream of COX, including PGES, the final enzyme in the conversion of PGE_2_ and the target receptors for PGE_2_ (EP_1–4_) were unaffected by age or ASA administration. This finding demonstrates that the increases observed in hypothalamic PGE_2_ in aging (Begg et al., 2012a,b) are not a function of PGES expression. The lack of differences observed on EP_1–4_ receptor expression may indicate that the changes observed in PGE_2_ levels are not great enough to produce changes in receptor regulation.

The data also indicate that iNOS may be involved in the reduced fluid intake in aging. Indeed, iNOS mRNA expression, but not nNOS or eNOS, was significantly upregulated in aged animals. This is expected given that NO modulates PGE_2_ during the inflammatory response. However, ASA treatment did not reduce iNOS expression. This is of note given that suppression of PGE_2_ generally results in concurrent NO suppression. Given that there is possible involvement of NOS in both fluid intake (Calapai et al., 1992) and sodium intake (Abrão Saad et al., 2004), the effect of central NOS inhibition in the aging rat may be of interest and may help determine the observed differences in ingestive behaviors in the current study. For example, reduced fluid intake in aging may be eicosanoid mediated in regions such as the median preoptic nucleus of the anterior hypothalamus, while increased NOS may reduce sodium intake acting in regions such as the lamina terminalis (Begg, 2017).

The results of this study indicate that the reduction of fluid intake following dehydration in aging is mediated by an increase in central eicosanoid production and that drinking can be restored by COX inhibition. They also demonstrate that the reduced sodium intake is independent of this pathway, with the NO system being a possible target for future research in this model.

## Data Availability Statement

The datasets generated for this study are available on request to the corresponding author.

## Ethics Statement

The animal study was reviewed and approved by La Trobe University Animal Ethics Committee.

## Author Contributions

DB, AS and RW designed the experiments. DB and RW collected and analyzed the data. DB wrote the manuscript. All authors reviewed and edited the manuscript.

## Conflict of Interest

The authors declare that the research was conducted in the absence of any commercial or financial relationships that could be construed as a potential conflict of interest.
